# Eyestrains among smartphone users while watching videos in Taipei MRT carriages: a comparison between sitting and standing postures

**DOI:** 10.1038/s41598-024-76334-9

**Published:** 2024-10-25

**Authors:** Yi-Lang Chen, Kuo-Hao Chu, Po-Chun Huang, Chieh-Ting Ho, Hong-Tam Nguyen

**Affiliations:** 1 Department of Industrial Engineering and Management, Ming Chi University of Technology, 84 Gung-Juan Road, Taishan, New Taipei, 243303 Taiwan; 2 Buy2sell Vietnam, Ho Chi Minh, 751000 Vietnam

**Keywords:** MRT carriages, Smartphone use, Eyestrain, Critical flicker fusion frequency (CFF), Visual fatigue scale (VFS), Viewing distance, Health care, Risk factors

## Abstract

In MRT carriages, more passengers are watching videos on their smartphones during daily commutes, yet the impact of these viewing conditions on visual strain remains unexplored. This study examined eyestrain in 24 male and 24 female participants while watching videos on smartphones in two postures (sitting and standing) and over two durations (15 min and 30 min) in Taipei MRT carriages. We measured critical flicker fusion frequency (CFF) reduction, visual fatigue scale (VFS) scores, and viewing distance (VD) for each trial. Results revealed significant effects of sex, posture, and viewing duration on visual strain, with notable two-way interactions (sex × posture and posture × time) influencing most outcomes. Women showed greater CFF reduction while sitting compared to standing (3.47 Hz vs. 1.90 Hz, *p* < 0.001) and maintained shorter VDs while seated (25.5 cm vs. 34.3 cm, *p* < 0.001). However, they reported higher VFS scores when standing (8.94 vs. 4.60, *p* < 0.001), indicating increased discomfort while standing in the moving MRT carriage. Men exhibited higher CFF reduction (2.81 Hz) than women while standing (1.90 Hz) (*p* < 0.01), but this difference was not observed while sitting. Eyestrain indicators significantly worsened after 30 min of viewing compared to 15 min, with stronger effects on VFS scores and VDs. This finding supports the recommendation to take breaks after 20 min of smartphone use, even in MRT carriages. Although subjective eyestrain is not always recognized, watching videos on smartphones while seated can result in unexpectedly high levels of objective visual fatigue, indicated by CFF reduction. Our findings suggest that eyestrain patterns in MRT carriages differ from those reported in previous studies. Seated passengers, particularly females with shorter VD, may experience unrecognized eyestrain that could impact eye health, while standing passengers may face greater discomfort. These results highlight the need for increased awareness and preventive measures to mitigate eyestrain in digital public transportation environments.

**Supplementary Information:**

The online version contains supplementary material available at 10.1038/s41598-024-76334-9.

## Introduction

Research indicates a growing global trend in spending time on daily activities and media entertainment via smartphones^1–3^. In Taiwan, as of 2023, the average daily smartphone usage per person is 7.23 h. Watching videos is one of the primary uses, with smartphones being the most commonly used device due to their mobility. The average daily video viewing time per person is 1.32 h, and this number continues to rise annually^4^. This is particularly popular among young people and can sometimes lead to addictive behaviors^5^. While smartphones have introduced numerous conveniences and advantages to modern lifestyles, they have also brought about persistent challenges.

One of the most critical challenges is neck and shoulder strain, which has been examined in relation to various factors such as user posture^6^, hand usage^7^, and participant demographics^8^. These studies have highlighted the distinct effects of these factors on head and neck flexion angles. Previous research has also explored eyestrain associated with smartphone use, primarily through epidemiological and optical analyses^9–12^. While some prior investigations have integrated gaze angle and viewing distance (VD) along with head and neck flexion angles^6,13–15^, the lack of direct indicators to evaluate visual fatigue leaves the assessment of eyestrain largely speculative.

Digital eyestrain can be categorized into external and internal symptoms, as outlined by Sheedy et al.^16^. Ergonomic studies on smartphone use typically focus on internal symptoms, such as visual fatigue and headaches, which result from strain on the eye’s refractive and binocular vision systems. A comprehensive assessment of these symptoms can benefit from both objective measures, such as reductions in critical flicker fusion frequency (CFF), and subjective measures, like the visual fatigue scale (VFS). CFF refers to the frequency at which a flickering light source appears steady, reflecting the connection between the eyes and brain^17^, and is widely accepted for evaluating eyestrain and visual fatigue. To measure subjective visual fatigue, researchers frequently use the VFS developed by Heuer et al.^18^, a well-established tool in visual fatigue studies^19–21^.

Although VD is an indirect indicator of visual strain, it remains a critical measure. Previous studies consistently show that shorter VDs increase the strain on accommodation and vergence, potentially exacerbating eyestrain symptoms^22–24^. Despite being aware of the risks, users often fail to maintain an appropriate VD while engaging with their smartphones. Ho et al.^25^ demonstrated the effectiveness of an application that reminds users to keep a safe distance from their devices, promoting healthier usage habits. To reduce eyestrain, maintaining a proper VD and limiting screen time are generally recommended^26–28^.

The Mass Rapid Transit (MRT) is a widely used and punctual form of public transportation in major cities. Over 40% of Taipei MRT passengers use smartphones^29^, with even higher rates reported in the Delhi Metro^30^. These data, however, were collected more than six years ago. Given the rapid advancements in personal video and entertainment software, current smartphone usage rates are likely even higher. Our recent field observations suggest that over 80% of Taipei MRT passengers, especially younger individuals, now use smartphones. In MRT carriages, watching videos tends to be more common than texting or browsing, as the confined and often crowded environment limits standing passengers to using only one hand. While video watching requires less active engagement than texting or browsing and may be more convenient during transit, the sustained attention it demands can increase the risk of visual strain, particularly in a moving environment like an MRT carriage. Wu et al.^31^ found that viewing dynamic content, such as films, significantly increases visual fatigue compared to static content. The unique interaction style of watching videos in MRT carriages presents challenges in assessing eyestrain^32^, potentially heightened by the moving environment^33^. These observations highlight the need for a comprehensive evaluation of eyestrain in such conditions to promote behaviors that mitigate visual strain. While previous research has investigated eyestrain associated with smartphone use^6,9–15^, there remains a notable gap in understanding how ergonomic factors related to visual load are influenced by environmental conditions, such as those present in transit settings.

This study aimed to investigate the impact of smartphone video watching on visual fatigue using direct objective and subjective measures, namely CFF reduction and VFS scores, respectively, along with the indirect indicator of VD. We hypothesized that fixating on a smartphone screen, especially during dynamic activities like standing in a relatively unstable environment, could potentially heighten eyestrain compared to sitting. This increase in visual load might result from the higher demands on the eyes’ accommodation and vergence while standing. Additionally, it was postulated that visual load would intensify with longer viewing times, as exemplified by the 30-min duration.

## Materials and methods

To assess the visual load when viewing videos on smartphones in MRT carriages, a group of participants (24 men and 24 women) engaged in simulated smartphone interactions for either 15–30 min, adopting either a sitting or standing posture. Data collection included reductions in CFF, self-reported VFS scores, and VD. These metrics were employed to evaluate the impact of mobile video viewing on eyestrain. This study received ethical approval from the Ethics Committee of National Taiwan University, Taiwan (Approval code: NTU-REC 202312EM051), affirming its adherence to ethical standards and guidelines. All testing processes were carried out in accordance with the relevant guidelines and regulations of the 2013 World Medical Association Declaration of Helsinki. Informed consent was obtained from all participants and attested for publication of the identifying information/images in an online open-access publication.

### Participants

In this study, we recruited 48 young Taiwanese participants, equally divided by sex, all of whom had no history of musculoskeletal disorders or visual impairments. The selection criteria required participants to have a minimum of one year of daily smartphone usage for at least 3 h, in line with previous research. Studies have shown that young students, both in Eastern regions^34^ and Western regions^35^, typically use their smartphones for at least 3 h per day. Among male participants, the average (± standard deviation) age, height, and body mass were 21.2 (± 1.3) years, 173.6 (± 4.6) cm, and 69.8 (± 9.8) kg, respectively. Female participants had an average age, height, and body mass of 21.7 (± 1.1) years, 160.2 (± 3.5) cm, and 53.8 (± 8.2) kg, as detailed in Table 1. Prior to data collection, participants received a thorough explanation of the testing protocol and provided informed consent using a consent form.

**Table 1:** Descriptive information of male and female participants in the study.

Items	Males (*n* = 24)	Females (*n* = 24)	Differences	*p*-value
Age (years)	21.2 ± 1.3 0.3	21.7 ± 1.1	-0.5	0.544
Stature (cm)	173.6 ± 4.6	160.2 ± 3.5	13.4	< 0.001***
Body mass (kg)	69.8 ± 9.8	53.8 ± 8.2	16.0	< 0.001***
Daily smartphone usage time (hours)	4.8 ± 1.6	4.5 ± 1.7	0.3	0.683
Critical fusion frequency (Hz)	38.8 ± 2.6	39.1 ± 1.7	-0.3	0.862

*Note* Data (mean ± standard deviation) were examined between sexes by independent t test; *** *p* < 0.001.

### The CFF measurement

The CFF is a well-recognized metric used to gauge eyestrain, where a reduction in CFF value corresponds to increased eye fatigue^36,37^. We measured CFF using a Handy Flicker device (Handy Flicker HF-II, Neitz, Tokyo, Japan). The ascending and descending thresholds were meticulously recorded and then averaged to establish the pre- and post-task CFF for each measurement. This process of measuring CFF was repeated twice, and the resulting values were averaged for precision.

During each test, the flicker frequency was systematically increased from a minimum threshold of 20 Hz until participants consistently perceived the presented light stimuli as stable. This threshold, as perceived by participants, provided insights into the critical frequency, indicating the highest frequency at which participants no longer perceived flickering. Following this, the frequency was gradually decreased until participants indicated that they once again perceived the presented light stimuli as flickering or vibrating, following the methodology outlined by Gautam and Vinay^17^.

### Subjective visual fatigue rating

In this study, the VFS score was used to assess the eyestrain after viewing the films in a requested testing condition^18^. The VFS questionnaire comprises six items: (1) It is hard for me to see (2). I have a strange feeling around my eyes (3). My eyes feel tired (4). I feel numb (5). I feel dizzy looking at the scree (6). I have a headache. Participants rated these questions on a 10-point scale, where 1 indicated “not at all” and 10 indicated “extremely serious”. The scores for the six items were then added up to provide an overall assessment of the severity of visual fatigue experienced^38^. In our analysis, we considered the change in the VFS resulting from the undertaken video viewing activity. Specifically, the fatigue score recorded at the beginning of each testing session was regarded as the baseline against which subsequent measurements were compared.

### The VD measurements

Since this study was conducted in real MRT carriages, it lacked the controlled environment of a laboratory. The VD measurements primarily followed the field observation protocol established by Chen et al.^6^. To mitigate this, we pre-selected a fixed position in the carriage for conducting the sitting and standing experiments. During the pilot test, we positioned a camera 2 m away from the vertical sagittal plane of the participant. We then used the camera’s controlled parameters to establish the dimensional ratio on the participant’s sagittal plane. After calibration, this ratio was employed as the basis for measuring the VD during the experiments, estimating the actual VD from the size obtained on the image. To derive VD, we meticulously captured symmetrical sagittal images and utilized CorelDRAW (Graphics Suite, 2023 v24.5, Corel Co., Ottawa, ON, Canada; URL Link: see Supplementary Material) for precise digital markings. The experimenter identified the participant’s eyeball and the midpoint of the phone’s length on the digital images to calculate VD^6,14,24^. In the pilot test, the discrepancy between actual and estimated VD was a mere 0.6 cm, showcasing an acceptable accuracy that underscored the quality of our estimations.

### Experimental design and procedure

This study collected data on CFF reduction, VFS scores, and VD measurements across four distinct smartphone-viewing trials. These trials combined two viewing postures (sitting and standing) and two viewing durations (15 min and 30 min) for 48 young participants. The 30-min viewing duration aligns with Leung et al.^1^, who asked participants to watch a movie on a smartphone while walking on a treadmill or sitting in a chair, though they did not measure intermediate times. In Taipei, the average one-way commuting time is 32.7 min, totaling about 1 h daily^39^. Given that the MRT is a public space, this study selected a section of the Taipei MRT during off-peak hours, using a predetermined zone within a carriage to conduct round-trip tests along a suitable route. Each half-day session consisted of up to six trials, with a maximum of two trials per participant, allowing for a maximum of three participants per session. To minimize errors and participant fatigue, a minimum 10-min resting period was required between trials for the same participant. Excluding holiday peak traffic periods, data collection was completed over approximately 20 workdays, resulting in 192 valid trials (48 participants × 2 postures × 2 time durations).

During the test, participants watched four films in varying combinations of posture and duration, with the sequence randomized. In each trial, CFF and VFS assessments were conducted at both the start and end of the viewing session. Participants used their own smartphones, which had the assigned movies pre-loaded for the experiment. The brightness of the smartphones was set to each participant’s usual preference. With most screens ranging between 6.1 and 6.8 inches and no significant size variations, we assumed that screen brightness and size did not significantly influence the testing outcomes.

To avoid distorting the testing situation compared to the real world, participants were allowed to carry their usual commuting backpacks. However, backpacks that were too large or heavy, potentially altering body posture, were excluded from the study. Once the test began, participants were instructed to watch the assigned videos in their most comfortable and natural posture, and to maintain that posture throughout the test. However, they were permitted to make slight adjustments if they felt uncomfortable. Each trial lasted either 15–30 min, with VD data recorded during the final 1-min interval at 15-s intervals. These values were averaged for analysis. The Taipei MRT carriage typically maintains a temperature of 24 °C, with an average speed of 35 km/hour and a maximum speed of 80 km/hour. The average illumination in the carriage at 100 cm above the floor is over 250 lx, with a minimum of 200 lx^40^.

### Statistical analysis

The collected data from the study underwent thorough analysis using SPSS 23.0 statistical software (IBM Corp., Armonk, NY, USA), with a significance level of 0.05 for all tests. The primary objective was to examine the impacts of participant sex (men and women), viewing posture (sitting and standing) and duration (15 min and 30 min) on the measured variables (CFF reduction, VFS, and VD) through a three-way ANOVA. In the analysis, participant sex was designated as a between-subject factor, while posture and duration were considered within-subject factors. Post-hoc comparisons were performed using independent t-tests to uncover significant differences between groups. To assess the practical significance of any identified independent variables, power values were calculated following Cohen’s established guidelines^41^. An effect size of ≥ 0.2 signifies a small effect, ≥ 0.5 represents a medium effect, and ≥ 0.8 indicates a large effect. Prior to conducting the analyses, the Kolmogorov-Smirnov test was utilized to evaluate the alignment of numerical variables with the normal distribution. Additionally, Levene’s test was employed to examine the equality of variances, ensuring the robustness of the analytical framework.

## Results

In our study, the male participants were significantly taller and heavier than the female participants (*p* < 0.001). However, baseline CFFs were consistent across both sexes, averaging 38.8 Hz for males and 39.1 Hz for females. Their CFF range aligned with the typical values reported for adults, which generally fall between 35 and 40 Hz^17^. This suggests that the variation in CFF reduction can be attributed to differences in testing conditions. The results of the Kolmogorov-Smirnov test indicated that the collected data, both for the entire group and subgroups, followed a normal distribution (*p* > 0.05). Similarly, Levene’s test demonstrated homogeneity in the data (*p* > 0.05). These findings confirmed that the data met the assumptions necessary for subsequent ANOVAs.

Table 2 presents the findings of the three-way ANOVA, showing the effects of participant sex, viewing posture, and viewing durations as independent variables. The analysis indicated a significant influence for almost all responses studied. However, due to the significant two-way interaction effects between sex and posture, as well as posture and time, the main effects of the independent variables require further cross-analyses for confirmation.

**Table 2:** Three-way ANOVA results for all responses.

Variables	Responses	MS	F	*p*-value	Power
Sex	CFF reduction	2.08	1.19	0.276	0.192
	Visual fatigue score	145.26	12.14	< 0.01**	0.934
	Viewing distance	3400.33	193.30	< 0.001***	1.000
Posture	CFF reduction	35.88	20.53	< 0.001***	0.995
	Visual Fatigue score	382.51	31.98	< 0.001***	1.000
	Viewing distance	1150.52	65.40	< 0.001***	1.000
Time	CFF reduction	66.51	38.05	< 0.001***	1.000
	Visual fatigue score	772.01	64.54	< 0.001***	1.000
	Viewing distance	468.75	26.65	< 0.001***	0.999
Sex × Posture	CFF reduction	24.08	13.78	< 0.001***	0.958
	Visual fatigue score	109.51	9.16	0.003**	0.853
	Viewing distance	841.69	47.85	< 0.001***	1.000
Sex × Time	CFF reduction	1.33	0.76	0.384	0.140
	Visual fatigue score	13.55	1.13	0.289	0.185
	Viewing distance	2.08	0.12	0.731	0.064
Posture × Time	CFF reduction	1.51	0.86	0.355	0.152
	Visual fatigue score	64.17	5.37	0.022*	0.635
	Viewing distance	82.69	4.70	0.031*	0.578
Sex × Posture × Time	CFF reduction	6.75	3.86	0.051	0.489
	Visual fatigue score	3.26	0.27	0.603	0.081
	Viewing distance	9.19	0.52	0.471	0.111

*Note* CFF, critical flicker fusion frequency; MS, mean square; F, F-value; In the analyses, all degrees of freedom = 1; * *p* < 0.05, ** *p* < 0.01, *** *p* < 0.001).

**Fig. 1:**
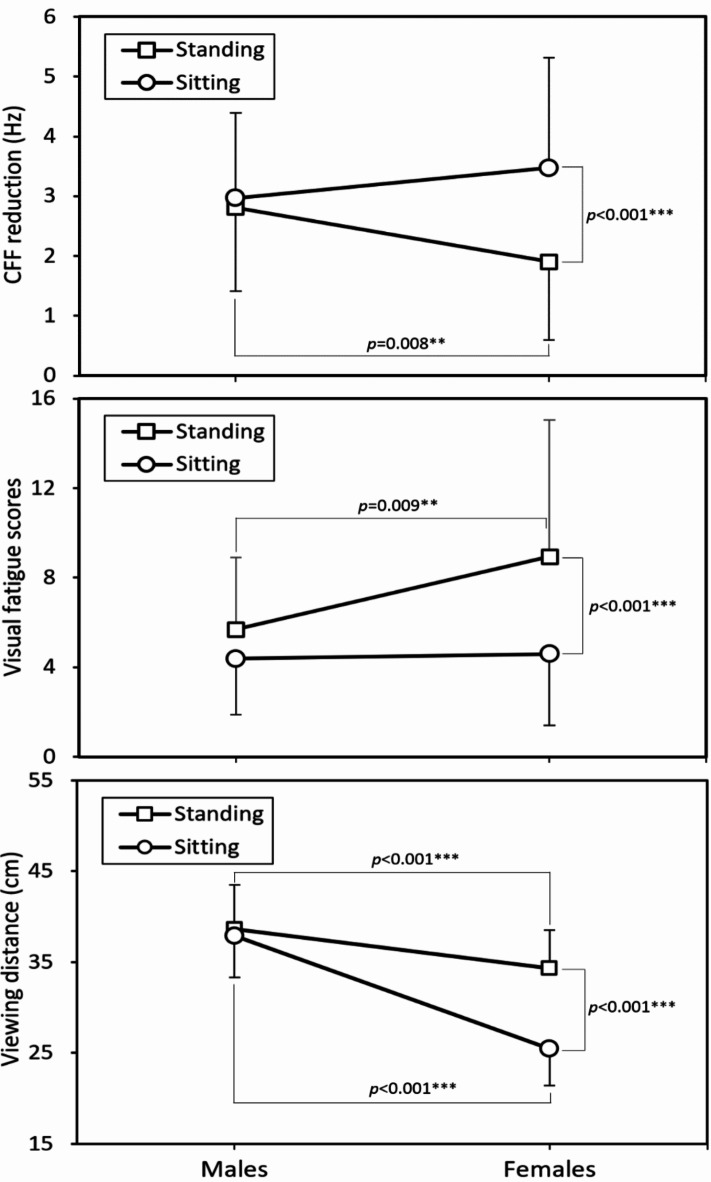
Interaction effects of sex × posture on the three investigated responses (The error bars around the mean values represent the positive or negative standard deviations; ** *p* < 0.01, *** *p* < 0.001).

**Fig. 2:**
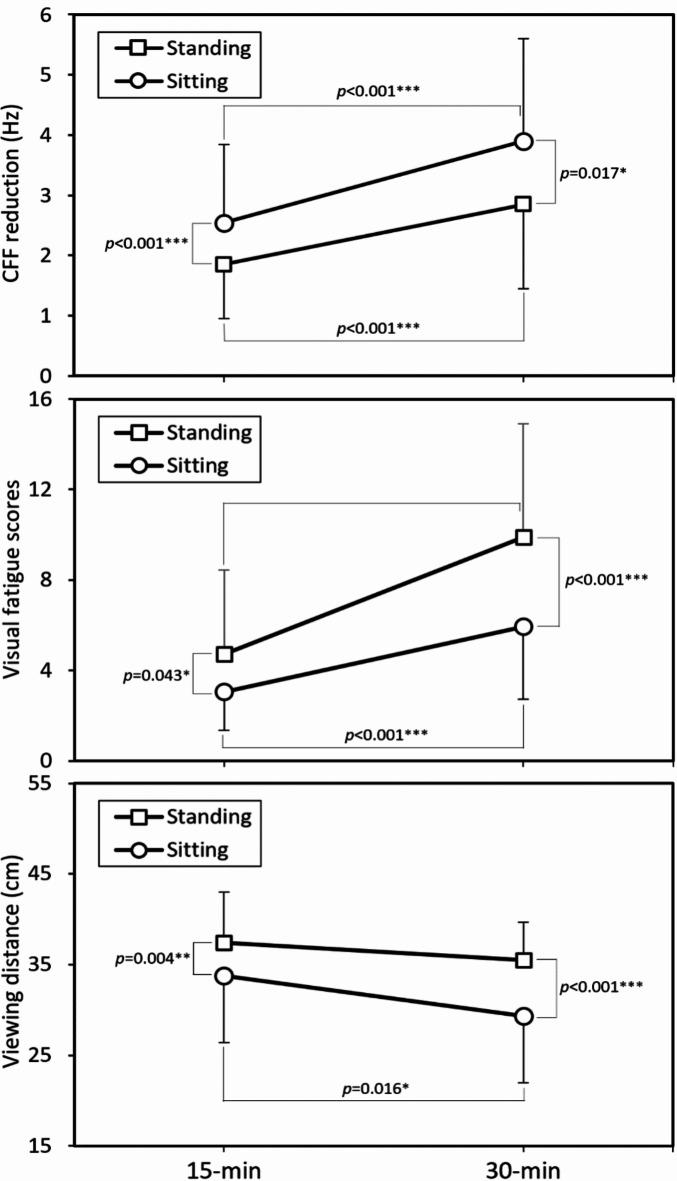
Interaction effects of posture × viewing time on the three investigated responses (The error bars around the mean values represent the positive or negative standard deviations; * *p* < 0.05, ** *p* < 0.01, *** *p* < 0.001).

Figure 1 compares three responses for two postures across genders. In general, men exhibited lower subjective fatigue scores and longer VD than women, with nonsignificant posture effects on the responses (all *p* > 0.05). Conversely, posture effects were significant for women (all *p* < 0.001). Women showed greater reductions in CFF and shorter VDs when sitting, but experienced lower subjective fatigue levels when standing. These opposing results warrant further discussion. Figure 2 illustrates the interaction between posture and time. As viewing time increased from 15 to 30 min, fatigue levels rose and VD decreased. Additionally, the differences in VFS score and VD between postures became more pronounced with the extension to 30 min.

## Discussion

The growing trend of watching videos on smartphones in MRT carriages necessitates an understanding of how various postures and viewing durations affect eyestrain, critical for user comfort and well-being. Unlike browsing or texting, which have been extensively studied^42–46^, watching videos presents unique challenges due to its continuous narrative, potentially limiting the relevance of previous research. Additionally, sustained viewing often occurs unconsciously. This insight led us to examine a critical yet often overlooked aspect of modern digital entertainment: the risk of eyestrain during smartphone video viewing in different postures and across sexes in MRT carriages. Our findings unexpectedly revealed that the visual load from watching videos while seated may not be lower than that experienced while standing in MRT carriages. Viewing posture affected both objective and subjective eyestrain differently between sexes, with prolonged viewing further amplifying the strain. This suggests that the visual load pattern of watching smartphone videos in MRT carriages differs from that observed in typical settings. Ignoring the impact of sitting and watching in a moving MRT carriage may lead to adverse effects on eye health, particularly for females, due to the significant shortening of VD. Meanwhile, standing posture can cause greater subjective discomfort. Thus, smartphone use in MRT carriages should be moderated, even when seated comfortably.

While watching videos in MRT carriages, women reported greater subjective eyestrain (VFS score) while standing (*p* < 0.001), whereas objective measurements (CFF reduction) showed increased strain when sitting (*p* < 0.001). Interestingly, the effect of posture on eyestrain was observed to be significant only in female participants (see Fig. 1), suggesting potential differences in smartphone viewing behaviors between sexes. The mechanisms behind this phenomenon are complex and may involve inherent sex differences in postural characteristics^46,47^ and viewing behavior^6,48–51^, particularly in specialized environments like MRT carriages. Our study revealed a potentially dangerous oversight: the assumption that sitting is less visually demanding may cause users to underestimate the strain on their eyes, especially females. While the objective CFF reduction indicated increased visual fatigue, the subjective VFS measure did not consistently reflect this strain in female participants (Fig. 1), suggesting an unrecognized visual load. These findings emphasize the need to reevaluate the habitual behavior of watching videos on smartphones in MRT carriages, particularly during extended viewing sessions, to mitigate eyestrain.

Figure 1 showed a consistent reduction in CFF across both viewing postures for male participants. In contrast, female participants exhibited a significant difference in CFF reduction between the two postures, which may be attributed to variations in VD. Generally, a shorter VD, especially on mobile devices, demands higher vergence and accommodation responses, leading to tension in the extraocular, ciliary, and pupillary muscles^25,52^. This tension is a primary factor in digital eyestrain^23,24^. Near vision tasks are unnatural for the eyes, which have evolved to focus on distant objects where the eye muscles are more relaxed^53^. Although there is limited research on differences in visual load when viewing smartphones while seated versus standing, several studies have investigated VD. For example, Chen et al.^6^ found almost no difference in VD between sitting and standing for both males and females. Similarly, an observational study by Boccardo^54^ reported average VDs of 37.4 cm for standing and 36.1 cm for sitting, with a difference of 1.3 cm. In line with these findings, our male participants showed a smaller difference in VD between postures (standing: 38.6 cm; sitting: 37.9 cm). However, in our study, this difference increased to 8.8 cm among female participants (standing: 34.3 cm; sitting: 25.5 cm). These variations in VD between the two viewing postures across sexes may be influenced by the specific environment of MRT carriages.

Sex differences in VD (see Fig. 1) may also be partly explained by variations in average body size, particularly the relatively shorter forearm length in females^54^, resulting in a shorter VD compared to males (Males: 38.6 cm; Females: 34.3 cm). However, this difference in VD does not imply that women are inherently more susceptible to visual fatigue than men. In fact, female participants experienced significantly less objective eyestrain while standing compared to male participants (1.90 vs. 2.81 Hz, *p* < 0.01), despite having a significantly shorter VD (*p* < 0.001). On the other hand, females exhibited a much shorter VD while sitting than when standing. Before the advent of PCs, smartphones, and tablets, the ideal reading distance was determined by the Harmon method, which involves making a fist, holding it to one’s cheek, and measuring the distance from the elbow to the eyes. For adults, this Harmon distance is typically 36-41 cm^25^. Engaging in near-vision tasks at distances shorter than this recommended VD range, such as reading and writing, can lead to eyestrain or headaches^55^. This range is similarly advised for smartphone use^25^. In Taiwan, the average difference in forearm length between sexes among youths is approximately 3.5 cm^56^, which corresponds with the observed sex difference in VD in the standing position (4.3 cm, Fig. 1), but not in the sitting position (12.4 cm). Females may tend to hold their phones closer to their eyes while sitting, which likely explains the shorter VD observed. This discrepancy emphasizes the unusually short VD and contributes to higher levels of objective eyestrain experienced by this group. Additionally, the relatively shorter VD observed in women in our study may also be attributed to postural differences between sexes. Women may tend to hold their phones closer to their eyes, resulting in a shorter VD and higher visual load (Fig. 1). Korakakis et al.^47^ found that women consistently adopt more upright postures than men in standing scenarios, which may also explain the significant difference in VD between the two viewing postures for female participants.

In the VFS results, females exhibited opposite trends in visual fatigue across the two smartphone viewing postures. VFS scores indicated higher subjective fatigue while standing, despite lower levels of objective fatigue in this posture. This suggests a potential conflict between perceived and actual eyestrain in different postural conditions. Visual fatigue, often caused by a mismatch between accommodation and convergence^57^, can lead to symptoms such as eye strain, difficulty focusing, headaches^58^, and motion sickness^59,60^. Moving visual environments are known to induce postural adjustments and motion sickness in healthy adults^61^. Moreover, smartphones are among the most commonly used devices for viewing dynamic visual content in moving vehicles^62,63^, such as MRT carriages. Previous research has thus advised avoiding visual activities that conflict with the brain’s physiological expectations, such as reading in a moving vehicle^33^. In our study, the higher levels of subjective fatigue observed in the relatively unstable standing position may be indicative of motion sickness, compared to sitting. Furthermore, real-life experience suggests that peripheral visual information, such as moving scenery outside the window, is likely more pronounced when standing than when sitting. Females have been reported to be more susceptible to motion sickness and experience greater discomfort than males^49–51^, which may also explain why female participants exhibited higher VFS scores compared to males when standing. However, since we did not measure motion sickness levels, these speculations, based solely on eyestrain measures, require further investigation in future studies.

In our analysis, extending smartphone viewing time from 15 to 30 min resulted in significant increases in both subjective and objective indicators of visual load, as well as a reduction in VD (Table 2, all *p* < 0.001). Table 2 also highlights the significant interaction effects of posture and viewing time on both VFS scores and VD (*p* < 0.05). Notably, the differences in VFS scores and VD between the two postures became more pronounced when viewing duration extended to 30 min (Fig. 2, *p* < 0.001). Specifically, greater subjective visual fatigue was reported while standing, whereas shorter VD was observed while sitting, indicating that the adverse effects of these postures were exacerbated with prolonged viewing. Additionally, the 20-20-20 rule recommends taking breaks every 20 min by looking at an object 20 feet away for at least 20 s^28,64^. This guideline suggests that 20 min may be a critical threshold for visual fixation tasks, reinforcing the need for periodic breaks to alleviate visual strain. Our findings on smartphone video viewing in MRT carriages align with the 20-20-20 rule, further supporting its role in mitigating visual fatigue.

This study has several limitations. Firstly, we recruited 48 young men and women as participants, limiting the generalizability of our findings to other demographic groups, such as children and the elderly, even though young individuals comprise the largest segment of smartphone users in Taipei MRT carriages. Secondly, the durations of smartphone viewing (15 and 30 min) used in our study do not reflect actual daily usage patterns, posing challenges in extrapolating our results to real-world scenarios. Moreover, the degree of smartphone usage addiction across different sexes was not rigorously controlled, and the selection of the videos used in this study might carry inherent limitations, warranting further exploration. Although all participants had normal (or corrected) vision, their eye-related parameters, such as vergence, saccades, and phorias, were not measured. This omission could potentially impact the results and should be acknowledged. During video viewing in MRT carriages, fluctuating environmental factors (including lighting and crowding of passengers around the users) and smartphone factors (including screen brightness and size) could influence visual fatigue, emphasizing the need to include these factors in future research. VD data was sampled and averaged during the final 1-min period after 15–30 min of viewing, which also represents a limitation of this study. Finally, it must be noted that this study was conducted in Taipei MRT carriages. The design and environment of MRT carriages in different regions and countries vary, which may also cause differences in study results.

## Conclusions

The increasing use of smartphones in MRT carriages has raised concerns about the visual strain associated with viewing screens in this unique, dynamic environment. This concern prompted us to conduct the present study, which aimed to evaluate the visual fatigue caused by watching videos on smartphones within Taipei’s MRT carriages. The independent variables included participant sex, viewing posture, and time duration. The unique environment of MRT carriages may negatively impact users’ behavior while looking at their smartphones. Overall, the study found that objective visual fatigue was higher in the sitting position than in the standing position, contrary to expectations. Additionally, the objective and subjective visual fatigue levels of women in different postures were exactly opposite, likely due to the interaction between shorter VD and VIMS. The study also showed that viewing videos for 30 min caused higher visual strain than viewing for 15 min, suggesting that the 20-20-20 rule for visual activity may also apply in MRT carriages. Contrary to common expectations, sitting posture did not alleviate eye strain, while standing posture increased subjective discomfort, particularly among female users. This may be attributed to the dynamic MRT environment, emphasizing the visual load associated with smartphone use in such settings. These findings highlight the need to address the potential risks of eyestrain in public transit environments and should be taken seriously.

## Electronic supplementary material

Below is the link to the electronic supplementary material.

Supplementary Material 1

## Data Availability

The datasets generated during and analysed during the current study are available from the corresponding author on reasonable request.
