# Supplementary material for: Eyestrains among smartphone users while watching videos in Taipei MRT carriages: a comparison between sitting and standing postures
Source: Sci Rep. 2024 Oct 25;14:25407. doi: 10.1038/s41598-024-76334-9 (PMC11511868; doi:10.1038/s41598-024-76334-9)
Supplement: Supplementary file 1 — Supplementary Material 1 [file 41598_2024_76334_MOESM1_ESM.docx]

**The URL Link of the CorelDRAW used in the study is as follows:**

<https://coreldraw.corel.com/?content=trial&otc_opt=C0004%3A1%2CC0002%3A1%2CC0003%3A1%2CC0005%3A0%2CC0001%3A1&_ics=1726842709735&irclickid=~90RNGDFypsrhi~7adelri-a27301QLDsvuknoef-2SIzwnhfb52S&_gl=1*15ll0zk*_gcl_aw*R0NMLjE3MjY4NDI3MTAuQ2owS0NRand1clMzQmhDR0FSSXNBRGRVSDUzdmFZZDYtNkVaWmNzb3JNeGc3M3FBQ3FhS1N4aUFYTklzcmsyY01hX0JES1pmcFRUTGI4d2FBbUVNRUFMd193Y0I.*_gcl_au*MTM2NzIwODQ0MC4xNzI2NzgyMzkw*_ga*MTg0OTgzNTYxNS4xNzI2NzgyMzkw*_ga_N9MCS9J69S*MTcyNjg0MjcwOS4yLjAuMTcyNjg0MjcwOS42MC4wLjA>.
